# A Cross-Sectional Study on Associations Between BDNF, CRP, IL-6 and Clinical Symptoms, Cognitive and Personal Performance in Patients With Paranoid Schizophrenia

**DOI:** 10.3389/fpsyt.2022.943869

**Published:** 2022-07-06

**Authors:** Egor Chumakov, Mariia Dorofeikova, Kristina Tsyrenova, Nataliia Petrova

**Affiliations:** ^1^Department of Psychiatry and Addiction, Saint-Petersburg State University, Saint Petersburg, Russia; ^2^Saint Petersburg Psychiatric Hospital No̱ 1 Named After P. P. Kashchenko, Saint Petersburg, Russia; ^3^Laboratory of Neurophysiology and Pathology of Behavior, Sechenov Institute of Evolutionary Physiology and Biochemistry, Saint Petersburg, Russia; ^4^Center for Molecular Neurobiology, Institute of Developmental Neurophysiology, University Medical Center Hamburg-Eppendorf, Hamburg, Germany

**Keywords:** schizophrenia, cognitive impairment, biomarkers, C-reactive protein (CRP), interleukin-6 (IL-6), brain-derived neurotrophic factor (BDNF)

## Abstract

**Background:**

Cognitive impairment is among the core dimensions in schizophrenia and is a significant predictor of everyday functioning in people with schizophrenia. Given the enormous burden of schizophrenia, the search for its clinically relevant biomarkers is essential. Researchers have been trying to elucidate factors of cognitive impairment as well as personal performance, but the search is still ongoing. The aim of the study was to search for associations between BDNF, CRP, IL-6 and clinical symptoms, cognitive and personal performance in patients with paranoid schizophrenia.

**Methods:**

A total of 86 patients (53.5% women, mean age 31.1 ± 6.5) with paranoid schizophrenia (F20.0; ICD-10) in remission were examined. Clinical and neuropsychological examination included the Positive and Negative Syndrome Scale, Personal and Social Performance Scale, Calgary Depression Scale for Schizophrenia and the Brief Assessment of Cognitive Function in Schizophrenia. IL-6, BDNF, CRP levels were determined in the patients' blood serum.

**Results:**

Cognitive impairment was revealed in 79.1% of patients and was more profound in patients with higher number of hospitalizations (*p* = 0.006). The average BDNF levels were 13.38 ± 15.84 ng/ml, CRP concentration was 2.09 ± 2.54 mg/l, and IL-6 levels were 12.14 ± 5.88 pg/ml. There were no differences in biomarker levels or BACS results in patients that had different antipsychotic therapy or differed in the presence of anticholinergic therapy. CRP levels were higher in patients with longer disease duration, lower age of onset, more impaired personal social performance and processing speed. IL-6 was higher in individuals with lower working memory scores. PANSS negative subscale score negatively correlated and PSP score positively correlated with most cognitive domains. A linear regression established that the first episode vs. multiple episodes of schizophrenia could statistically significantly predict personal and social performance and cognition, including speech fluency and planning, as well as CRP levels.

**Conclusions:**

This study continues the search for biomarkers of schizophrenia and cognitive impairment in schizophrenia to improve the reliability of diagnosing the disorder and find new treatment approaches. The role of the number of psychoses experienced (first episode vs. multiple episodes of schizophrenia) in cognition, personal and social performance and inflammation is shown.

## Introduction

Schizophrenia is a heterogeneous disorder whose core features include positive, negative, and cognitive symptoms in addition to social and occupational dysfunction (1). Given the enormous burden of schizophrenia, the search for its clinically relevant biomarkers is essential (2). Since cognition is a significant predictor of everyday functioning in people with schizophrenia, including occupational disability and independent living skills (3–5), researchers have been trying to elucidate factors of cognitive impairment as well as personal performance, but the search is still ongoing.

An increasing number of clinical, epidemiological, and experimental studies have shown links between schizophrenia and inflammatory conditions (6–10). Inflammation is currently considered a potential mechanism in the development and progression of schizophrenia (6, 11) as well as other psychiatric disorders such as depression and bipolar disorder (7, 10, 12), with interleukin-6 (IL-6) being one of the most consistently elevated cytokines (13). It plays a role in the balance of pro- and anti-inflammatory responses, and its excessive levels are associated with neurodegeneration, as well as primary and enduring negative symptoms (deficit syndrome) of schizophrenia (2, 14). Plasma IL-6 was observed to partly mediate the association between higher childhood trauma scores and lower emotion recognition performance (impaired social cognition) (15). Association of IL-6 with lower recall span and digit coding has been found (16). Contradictory data have been published concerning IL-6 increase in first-episode psychosis and acute relapse, and positive correlations between IL-6 level and illness duration, depressive symptoms and worse mental and physical wellbeing have been proposed (17).

C-reactive protein (CRP) has been widely studied in different psychoses. Is has been associated with higher positive symptoms, as well as elevated body mass index and younger age (18). Recent meta-analysis showed that elevated CRP levels were related to all cognitive domains except for fluency (19). CRP levels may influence treatment choice, supporting precision medicine, with anti-inflammatory agents being tested in high inflammation subgroup of patients (2). Although currently no validated markers exist for schizophrenia patient stratification or the prediction of treatment efficacy, several authors (20) propose the use of inflammatory markers for diagnostic purposes and the development of novel therapeutic approaches.

Results concerning brain-derived neurotrophic factor (BDNF) levels and their factors (such as substance use) in schizophrenia have been partly contradictory. Although BDNF is not a disease-specific biomarker, it is considered as a potential biomarker having a very modest but significant relationship to cognitive functioning in schizophrenia, especially in chronic samples, that might be mediated by stress, early life trauma, insomnia and loneliness (19). Overall, no relationship between BDNF levels and either positive or negative symptoms has been found (18), and small effect sizes of correlations suggest that inflammation and decreased BDNF levels do not play a major role in cognitive dysfunction in most patients with schizophrenia (19).

Our goal was to search for associations between BDNF, CRP, IL-6 and clinical symptoms, cognitive and personal performance in patients with paranoid schizophrenia to try to identify specific subtypes of patients and search for their potential therapeutic targets.

## Materials and Methods

### Subjects

This cross-sectional study was approved by the local ethics committee (approval protocol #10562/1, 18.11.2020) and conducted in accordance with the Declaration of Helsinki. Patients were recruited from outpatient facilities affiliated with the Saint-Petersburg State University in 2021. Informed consent was obtained for all patients. Data were collected by trained psychiatrists 0.86 patients with paranoid schizophrenia [F20.0 in International Classification of Diseases (ICD)-10], including 46 (53.5%) women, were examined at the age of 31.1 ± 6.5 years at the stage of remission or previously, if all the criteria for remission (Supplementary Table 1) (21) were met, except time (6 months). Inclusion criteria were: age between 18 and 50 years. Exclusion criteria for all participants were: any comorbid psychiatric disorders or organic brain pathology, substance abuse throughout life, the presence of acute inflammation (diagnosed by a clinical interview and physical examination), chronic autoimmune diseases or use of anti-inflammatory drugs in the previous month. Only patients with paranoid schizophrenia were included to minimize the initial heterogeneity in clinical symptoms. Age range was chosen to minimize the probability of age-related cognitive decline and on the basis of methods' restrictions.

### Clinical and Neuropsychological Examination

Positive, negative and general symptomatology was assessed by one experienced psychiatrist with the Positive and Negative Syndrome Scale (PANSS) (22). Personal and Social Performance Scale (PSP) has been used to assess socially useful activities, personal and social relationships, self-care, and disturbing and aggressive behaviors (23). Depressive symptoms were evaluated with the use of the Calgary Depression Scale for Schizophrenia (CDSS) (24). Russian version of the Brief Assessment of Cognitive Function in Schizophrenia (BACS) was used to assess verbal memory, working memory, motor speed, verbal fluency, attention, and executive functioning (25). The primary measures for each test in the BACS was standardized by creating z-scores dependent on sex and age. The Scale for the Assessment of Negative Symptoms (SANS) was used in addition to PANSS to assess negative symptoms. The original SANS structure consists of nineteen items which are further divided into five subscales including affective flattening, alogia, avolition-apathy, anhedonia-asociality and attention. The SANS is recognized as a high-quality tool for negative symptom assessment (26), with good validity and reliability (27).

### Laboratory Methods

Five milliliters of blood were drawn from each subject by venipuncture into a vacuum tube without anticoagulant. Blood was immediately centrifuged at 3,000 g for 10 min, and serum was kept frozen at −70°C until analysis. Subjects were not fasting. IL-6 levels were determined using an automatic immunochemical analyzer Elecsys 2010 from Roche, Switzerland with a measuring range of 1.5–5,000 pg/mL. BDNF was determined in the blood serum of patients using quantitative enzyme-linked immunosorbent assay (R&D systems, USA). CRP levels were determined in blood serum using an “Architect c8000” automatic analyzer from Abbott, USA. CRP levels were determined using the immunoturbidimetric method and monoclonal antibodies to the protein detected by the ultrasensitive method with Abbott reagents (USA). The correctness of the determination of all the analytes was monitored using external quality control systems of laboratory studies of FSSVO (Russia) and RIQAS (UK).

### Statistical Analysis

Data analysis was performed using Microsoft Office Excel 2007 and statistical software package SPSS Statistics 23.0 (Statistical Package for the Social Sciences, version 23.0). The sample was divided into two groups: patients with a first episode of schizophrenia (FES; *n* = 50) and patients with multiple episodes of schizophrenia (MES; *n* = 36). Descriptive statistics were used, the Mann-Whitney test for independent samples, as well as the Kruskal-Wallis test for comparison between more than 2 groups. Variables with a continuous distribution were described as average and standard deviation (M ± σ); discrete variables and ordered data were described as the median and the 1–3rd quartiles [Md (Q1; Q3)]. Discrete indicators were described by the absolute value and fraction of the integer *n* (%). Correlation analyses were performed using the Spearman's test, and a linear regression analysis was used to define significant predictive factors. Statistical differences at *p* < 0.05 were considered significant.

## Results

### Sample Characteristics

The age of patients was 31.1 ± 6.5 years, and average duration of schizophrenia was 7.4 ± 8.0 years. Among the patients, there were 30 (34.9%) people with college level degrees and 26 (30.2%) with secondary vocational education 0.31 (36.0%) patient was currently studying or employed, and they had significantly higher PSP score (72.1 ± 7.8 vs. 63.8 ± 12.2, *p* = 0.018). Socio-demographic characteristics are presented in Table 1.

**Table 1:** Socio-demographic characteristics.

	**FES (*n* = 50)** ***n* (%)**	**MES (*n* = 36)** ***n* (%)**	***x*^2^(df); p**
Sex			*x*^2^(1) = 0.013; *p =* 0.911
Female	27 (54.0%)	19 (52.8%)	
Male	23 (46.0%)	17 (47.2%)	
Age, Md [Q1; Q3]; U; p	27.5 [24.0; 33.0]	35.0 [32.25; 38.0]	*U* = 336.5; *p* <0.001
Education			*x*^2^(2) = 0.459; *p =* 0.794
Secondary education	17 (34.0%)	10 (27.8%)	
Secondary vocational education	14 (28.0%)	12 (33.3%)	
College level degrees	19 (38.0%)	14 (38.9%)	
Work/study	23 (46.0%)	8 (22.2%)	*x*^2^(1) = 5.133; *p =* 0.023
Disability	6 (12.0%)	28 (77.8%)	*x*^2^(1) = 37.883; *p* <0.001

*FES, first episode of schizophrenia; MES, multiple episodes of schizophrenia*.

Eighty-six percentage of patients were treated with atypical antipsychotics, and 11.6% received the first-generation antipsychotic 0.2 (2.3%) patients were treated with a combination of them 0.23.3% of patients received anticholinergic drugs, 27.3%—antidepressants or mood stabilizers, and 6.8%—benzodiazepines. The latter were more frequently prescribed to older patients (38.7 ± 3.1 vs. 31.6 ± 6.9 years old, *p* = 0.047).

The patients' clinical characteristics are presented in Table 2. Family history of mental disorders (schizophrenia spectrum or affective) was found in 38.4% of patients. All patients received antipsychotic therapy mostly with second-generation antipsychotics.

**Table 2:** Clinical characteristics.

	**FES (*n* = 50)** **Md [Q1; Q3]**	**MES (*n* = 36)** **Md [Q1; Q3]**	**U; p**
Age of onset, years, M ± SD	26.0 [22.0; 32.0]	19.5 [16.0; 25.0]	*U* = 407.0; *p* <0.001
Disease duration, years	1.5 [1.0; 3.0]	11.0 [9.0; 21.0]	*U* = 0; *p* <0.001
Number of hospitalizations			
1–3	50 (100.0%)	0	
4–10	0	18 (50.0%)	
>10	0	18 (50.0%)	
Family history of mental disorders; *n* (%)	15 (30.0%)	18 (50.0%)	*x*^2^(1) = 3.540; *p =* 0.059*
PANSS			
Positive scale	12.0 [9.0; 15.0]	11.0 [10.25; 14.0]	*U* = 880.0; *p =* 0.860
Negative scale	14.0 [10.75; 16.0]	15.0 [11.25; 17.75]	*U* = 686.5; *p =* 0.060
General psychopathology scale	26.0 [19.0; 31.25]	27.0 [22.25; 31.75]	*U* = 792.0; *p =* 0.343
PANSS total	62.5 [58.0; 71.5]	63.0 [53.0; 80.0]	*U* = 842.5; *p =* 0.614
CDSS	1.0 [0.0; 3.0]	0.0 [0.0; 2.0]	*U* = 749.5; *p =* 0.154
PSP	71.0 [62.5; 79.0]	63.0 [52.0; 69.0]	*U* = 126.0; *p =* 0.012
SANS affective flattening	9.5 [5.75; 12.0]	9.5 [0.5; 14.0]	*U* = 199.5; *p =* 0.825
SANS alogia	5.0 [2.75; 6.0]	7.5 [6.0; 10.75]	*U* = 94.5; *p =* 0.003
SANS avolition-apathy	5.0 [3.75; 7.0]	5.0 [4.0; 8.75]	*U* = 175.5; *p =* 0.393
SANS anhedonia-asociality	6.0 [4.75; 8.25]	5.5 [2.5; 8.0]	*U* = 182.0; *p =* 0.498
SANS attention	3.3 [3.0; 4.25]	4.0 [4.0; 4.75]	*U* = 145.0; *p =* 0.094
Antipsychotic monotherapy; *n* (%)	35 (70.0%)	23 (63.9%)	*x*^2^(1) = 0.356; *p =* 0.551*
Antipsychotic therapy, *n* (%)			*x*^2^(1) = 3.267; *p =* 0.195*
Second generation antipsychotic	45 (90.0%)	29 (80.6%)	
First generation antipsychotic	5 (10.0%)	5 (13.9%)	
Combination of several antipsychotics	0 (0.0%)	2 (5.5%)	

*x^2^(df); p. FES, first episode of schizophrenia; MES, multiple episodes of schizophrenia; PANSS, Positive and Negative Syndrome Scale; CDSS, Calgary Depression Scale for Schizophrenia; PSP, Personal and Social Performance Scale; SANS, Scale for the Assessment of Negative Symptoms.

Only 18 (20.9%) patients in the sample had normal total BACS T-score (≥40) (Table 3). The most widespread cognitive deficit in the sample was observed in processing speed (84.9% of patients), and planning abilities were the most intact [normal in 48 (55.8%) patients].

**Table 3:** Cognitive functioning according to BACS assessment.

	**FES (*n* = 50)**	**MES (*n* = 36)**	***P***
Normal total BACS T-score; *n* (%)*	13 (26.0%)	5 (13.9%)	*x*^2^(1) = 1.855; *p =* 0.173
Total BACS T-score; Md [Q1; Q3]	28.5 [21.75; 40.0]	19.0 [7.0;30.75]	*U* = 625.0; *p =* 0.016
Normal verbal memory BACS T-score; *n* (%)*	18 (36.0%)	11 (30.6%)	*x*^2^(1) = 0.277; *p =* 0.598
Verbal memory BACS T-score; Md [Q1; Q3]	35.5 [24.75; 43.25]	32.0 [27.0; 41.0]	*U* = 797.5; *p =* 0.369
Normal working memory BACS T-score; *n* (%)*	11 (22.0%)	8 (22.2%)	*x*^2^(1) = 0.027; *p =* 0.869
Working memory BACS T-score; Md [Q1; Q3]	35.0 [25.5; 39.0]	29.0 [13.25; 39.0]	*U* = 748.0; *p =* 0.183
Normal motor skills BACS T-score; *n* (%)*	31 (62.0%)	13 (36.1%)	*x*^2^(1) = 7.203; *p =* 0.007
Motor skills BACS T-score; Md [Q1; Q3]	42.5 [32.75; 52.0]	36.0 [29.75;43.0]	*U* = 651.0; *p =* 0.029
Normal speech fluency BACS T-score; *n* (%)*	27 (54.0%)	11 (30.6%)	*x*^2^(1) = 4.665; *p =* 0.031
Speech fluency BACS T-score; Md [Q1; Q3]	40.5 [32.5; 50.0]	34.5 [25.5; 41.5]	*U* = 631.0; *p =* 0.019
Normal processing speed BACS T-score; *n* (%)*	9 (18.0%)	4 (11.1%)	*x*^2^(1) = 0.774; *p =* 0.379
Processing speed BACS T-score; Md [Q1; Q3]	26.0 [14.0; 36.25]	24.0 [13.5; 28.0]	*U* = 765.0; *p =* 0.237
Normal tower of London BACS T-score; *n* (%)*	34 (68.0%)	14 (38.9%)	*x*^2^(1) = 7.192; *p =* 0.007
Tower of London BACS T-score; Md [Q1; Q3]	47.5 [37.0; 62.5]	35.0 [5.5; 48.75]	*U* = 513.5; *p =* 0.001

^*^Normal BACS T-score ≥40; FES, first episode of schizophrenia; MES, multiple episodes of schizophrenia; BACS, brief assessment of cognitive function in schizophrenia.

When analyzing the entire sample of patients surveyed, it was found that women performed better than men in the token motor task (42.6 ± 14.6 vs. 35.0 ± 16.3, *p* = 0.026) and the Tower of London test (45.2 ± 20.1 vs. 28.7 ± 31.1, *p* = 0.016). Women also had more depressive symptoms (2.24 ± 2.62 vs. 1.05 ± 1.85, *p* = 0.025) and a better PSP score (70.6 ± 12.4 vs. 63.6 ± 10.1, *p* = 0.041).

The average BDNF levels in the sample were 13.38 ± 15.84 ng/ml, CRP concentration was 2.09 ± 2.54 mg/l, and IL-6 levels were 12.14 ± 5.88 pg/ml. There were no differences in biomarker levels or BACS results in patients that had different antipsychotic therapy or differed in the presence of anticholinergic therapy. Patients that were taking antidepressants or mood stabilizers had higher CRP levels (3.78 ± 2.89 mg/l vs. 1.34 ± 1.23 mg/l, *p* = 0.004), lower speech fluency (T-score of 33.2 ± 10.5 vs. 44.7 ± 11.4, *p* = 0.005) and processing speed (21.6 ± 15.6 vs. 32.8 ± 13.1, *p* = 0.037) that contributed to a significantly lower total BACS score (22.7 ± 10.9 vs. 34.6 ± 15.4, *p* = 0.013).

When analyzing the entire sample of patients surveyed, correlation analyses showed that total BACS score was negatively correlated with disease duration (*r* = −0.270, *p* = 0.012) and positively associated with the age of onset of schizophrenia (*r* = 0.245, *p* = 0.023). Significant correlations were found between those clinical features and results of the Tower of London (planning abilities, *r* = −0.407, *p* < 0.001 and *r* = 0.307, *p* = 0.004). Processing speed was also higher in patients with the later disease onset (*r* = 0.217, *p* = 0.045).

Depressed patients had significantly lower processing speed during symbol coding task (*r* = −0.414, *p* < 0.001). PANSS negative subscale score negatively correlated with most cognitive domains: total BACS score (*r* = −0.369, *p* < 0.001), verbal memory (*r* = −0.274, *p* = 0.011), working memory (*r* = −0.227, *p* = 0.035), motor skills (*r* = −0.316, *p* = 0.003), and planning and problem solving (*r* = −0.379, *p* < 0.001), and PSP score positively correlated with most cognitive functions (Table 4). SANS results had many significant correlations with BACS scores, especially because SANS “attention” subscale reflects cognitive functioning.

**Table 4:** Correlation analysis of associations between BACS scores in clinical scales scores in the sample.

		**BACS total**	**Verbal memory**	**Working memory**	**Motor skills**	**Speech fluency**	**Processing speed**	**Tower of London**
Negative symptoms, PANSS	Correlation coefficient	−0.369**	−0.274*	−0.227*	−0.316**	−0.106	−0.194	−0.379**
	Sig. (2-tailed)	0.000	0.011	0.035	0.003	0.333	0.073	0.000
PSP score	Correlation coefficient	0.595**	0.369*	0.189	0.406**	0.484**	0.540**	0.452**
	Sig. (2-tailed)	0.000	0.015	0.225	0.007	0.001	0.000	0.002
SANS affective flattening	Correlation coefficient	−0.333*	−0.152	−0.095	−0.404**	0.033	−0.439**	−0.245
	Sig. (2-tailed)	0.031	0.338	0.550	0.008	0.835	0.004	0.117
SANS alogia	Correlation coefficient	−0.527**	−0.389*	−0.602**	−0.265	−0.641**	−0.105	−0.479**
	Sig. (2-tailed)	0.000	0.011	0.000	0.089	0.000	0.506	0.001
SANS avolition-apathy	Correlation coefficient	−0.590**	−0.428**	−0.397**	−0.418**	−0.216	−0.482**	−0.524**
	Sig. (2-tailed)	0.000	0.005	0.009	0.006	0.169	0.001	0.000
SANS anhedonia-asociality	Correlation coefficient	−0.322*	−0.205	0.002	−0.349*	0.028	−0.568**	−0.260
	Sig. (2-tailed)	0.038	0.192	0.991	0.024	0.858	0.000	0.096
SANS attention	Correlation coefficient	−0.492**	−0.380*	−0.313*	−0.357*	−0.358*	−0.523**	−0.399**
	Sig. (2-tailed)	0.001	0.013	0.044	0.020	0.020	0.000	0.009

PANSS, positive and negative syndrome scale; PSP, personal and social performance scale; SANS, scale for the assessment of negative symptoms. *p < 0.05, **p < 0.01.

CRP levels were higher in patients with longer disease duration (*r* = 0.317, *p* = 0.003), lower age of onset (*r* = −0.307, *p* = 0.004), more impaired personal social performance (*r* = −0.313, *p* = 0.041) and processing speed (*r* = −0.254, *p* = 0.018). IL-6 was higher in individuals with lower working memory scores (*r* = −0.217, *p* = 0.044).

### Markers of the First-Episode Schizophrenia

The sample was divided into two groups: patients with a first episode of schizophrenia (FES; *n* = 50) and patients with multiple episodes of schizophrenia (MES; *n* = 36). MES patients were older and their social adjustment was worse. Antipsychotic medication sometimes was combined with mood stabilizers or antidepressants (10.0% of FES; 19.4% of MES), anticholinergic drugs (22.0% of FES; 25.0% of MES), and benzodiazepines (8.3% of MES).

Laboratory tests results in comparison groups FES and MES are presented in the Table 5. Two MES patients showed CRP levels above 10 mg/l, reflecting an active inflammatory response, while in 18 (20.9%) patients the level of CRP (3–10 mg/l) indicated the presence of systemic vascular inflammation. In the FES group, BDNF ranged from 0.23 to 101.81, whereas in the MES group the range was 1.21–46.13.

**Table 5:** Laboratory tests results in FES and MES patients.

	**FES (*n* = 50)**	**MES (*n* = 36)**	***U*; *p***
CRP; mg/l; Md [Q1; Q3]	0.99 [0.41; 2.33]	1.69 [0.63; 3.89]	*U* = 622.0; *p =* 0.015
CRP (3–10 mg/l); *n* (%)	7 (14.0%)	11 (30.6%)	*x*^2^(1) = 3.466; *p =* 0.063
IL-6; pg/ml; Md [Q1; Q3]	1.1 [0.88; 1.33]	1.15 [1.0; 1.4]	*U* = 753.5; *p =* 0.198
BDNF; ng/ml; Md [Q1; Q3]	8.85 [7.29; 12.15]	8.86 [6.68; 9.72]	*U* = 818.5; *p =* 0.476

*FES, first episode of schizophrenia; MES, multiple episodes of schizophrenia; CRP, C-reactive protein; IL, interleukin; BDNF, brain-derived neurotrophic factor*.

Correlation analysis revealed no association between biomarker levels and BACS scores or the severity of positive and negative PANSS symptoms in both FES and MES patients. IL-6 levels significantly correlated with CRP levels in FES patients (*r* = 0.372, *p* = 0.008), but this was not observed in MES patients.

A linear regression established that the FES but not CRP levels could statistically significantly predict BACS results [*F*_(2, 83)_ = 3.323, *p* = 0.041], the overall model fit was *R*^2^ = 0.074 (FES B = 19.83 ± 3.98, *p* = 0.031; CRP B = 0.57 ± 0.84, *p* = 0.497). PSP was predicted similarly: *F*_(2, 40)_ = 5.430, *p* = 0.008, *R*^2^ = 0.214 (FES B = 6.92 ± 3.40, *p* = 0.048; CRP B = −1.45 ± 0.81, *p* = 0.080). Among cognitive functions, speech fluency was predicted by FES: *F*_(2, 83)_ = 3.222, *p* = 0.045, *R*^2^ = 0.072 (FES B = 6.94 ± 2.91, *p* = 0.019; CRP B = −0.10 ± 0.57, *p* = 0.859), as well as planning: *F*_(2, 83)_ = 5.898, *p* = 0.004, *R*^2^ = 0.124 (FES B = 19.77 ± 5.80, *p* = 0.001; CRP B = 0.60 ± 1.13, *p* = 0.601). CRP levels themselves turned out to be influenced by the stage of disease: *F*_(1, 84)_ = 6.954, *p* = 0.010, *R*^2^ = 0.076 (FES B = −1.42 ± 0.54, *p* = 0.010).

## Discussion

The present study further contributes to the accumulation of data on the association of inflammatory biomarkers with the presence and severity of cognitive impairment in patients with schizophrenia at different stages of the disease. The study revealed that patients with FES had significantly lower CRP levels, better personal functioning and BACS results, including motor skills, speech fluency and planning, than patients with MES.

Although BDNF is likely the best-studied neurotrophin both in basic research and in relation to various clinical syndromes, studies on the concentrations of BDNF serum levels in patients with schizophrenia have had contradictory results (28). BDNF has attracted great interest as a possible biomarker because of its key role in synaptic remodeling during cognitive processes (29), in neurogenesis and neuroplasticity, and in the modulation of several neurotransmitter systems including the dopaminergic system involved in the pathophysiology of schizophrenia (30). In this regard, BDNF relationship with cognitive dysfunction in patients with different stages of psychosis, including but not limited to chronic schizophrenia, accumulates growing evidence (31).

The meta-analysis found minimal correlations between peripheral BDNF and neurocognitive phenotypes in people with schizophrenia, but effects for the reasoning and problem-solving domains were significant; thus, higher levels of BDNF expression corresponded to better performance on reasoning/problem-solving tasks (32). More recent studies found that patients with schizophrenia with lower BDNF concentrations were characterized by lower processing speeds (11, 32), cognitive impairment in visual learning and working memory domains (33), and executive function deficit (34). While another study aimed to explore the association between serum BDNF and cognitive functions in first-episode drug-naïve patients with schizophrenia found no significant associations between BDNF and cognitive performance (35). In our study we did not find an association between BDNF levels and cognitive function regardless the stage of the disease.

IL-6 has been widely studied in different aspects of schizophrenia (its onset, progression, association with different clusters of symptoms) (17). A meta-analysis has pointed out that IL-6 is increased in first-episode psychosis and acute relapse, and can be used as a state marker of schizophrenia (36). Recent meta-analysis shows elevated levels of IL-6 in the serum of patients with medication-naïve first episode psychosis (37). In a later study it was found that patients with schizophrenia had significantly higher IL-6 levels compared to the control group (38). However, previous studies have presented conflicting results regarding the levels of IL-6 in schizophrenia in connection to neurocognition. Some authors revealed no association of IL-6 elevation with general cognitive abilities in patients with schizophrenia (39). A study on neurocognition and daily functioning in relation to inflammation in individuals with schizophrenia did not find association of poor neurocognition and peripheral IL-6 levels (40). In contrast, relationship of serum IL-6 and cognitive functions in patients with schizophrenia was shown (16, 41). In our study, only the correlation of weak strength between IL-6 levels and lower working memory BACS scores was revealed in patients with schizophrenia regardless of the disease stage. In this regard, it is not possible to draw a convincing conclusion about the connection of cognitive functioning in schizophrenia with IL-6 levels on the data obtained. At the same time, the association of IL-6 levels with CRP in FES patients leaves the possibility of continuing research on this biomarker in larger samples.

Among biomarkers of inflammation potentially contributing to the development of cognitive impairment in schizophrenia, the evidence is more robust for CRP (42–44), with only a few studies that do not support this conclusion (39). Studies have established various associations of elevated CRP levels with cognitive domains. Thus, in one study elevated CRP patients with schizophrenia displayed significantly worse working memory and CRP was inversely correlated with cortical thickness in frontal, insula, and temporal brain regions (45), while in another study increased CRP level was mildly associated with worse performance in attention and with reduced cortical thickness in the caudal middle frontal, the pars opercularis and the posterior cingulate cortices—three regions related to attention (46). A systematic review of cytokines and CRP alterations with respect to cognitive impairment in schizophrenia summarized that most consistent results indicate worse cognitive performance in schizophrenia patients with higher CRP levels (43). A recent meta-analysis of cross-sectional studies of serum and plasma CRP levels in schizophrenia compared to healthy subjects found that the extent of the increase in peripheral CRP levels paralleled the increase in severity of positive symptoms, but was unrelated to the severity of negative symptoms (18). The significant association between measures of functional impairment and elevated CRP was detected in a group of stable outpatients with schizophrenia (47). In our study we found CRP levels association with impaired personal social performance and lower processing speed BACS scores, but the strength of association was weak. Moreover, FES but not CRP levels predicted cognitive functioning and social functioning in the examined patients with the emphasis on speech fluency and planning. CRP levels themselves turned out to be influenced by the stage of disease and were higher in patients with longer disease duration. In our study, CRP levels were higher in more old patients with chronic schizophrenia, while a recent meta-analysis showed that CRP levels did not change between the first episode of psychosis and with progression of schizophrenia (18).

A critical issue is whether the differences in cytokines and BDNF levels between patients and controls can be attributed to schizophrenia itself or whether they are attributable to confounding factors, particularly the possible effects of chronic antipsychotic medication (38). Although previously it was discussed whether cognitive impairment in schizophrenia is partly associated with long-term antipsychotic medication, the meta-analysis confirmed the existence of significant cognitive impairments at the early stage of schizophrenia in the absence of antipsychotic medication (48). No association was detected in a meta-analysis between initiation of antipsychotic medication notwithstanding whether these were typical or atypical antipsychotics and CRP levels elevation (18). In our study we also did not find an association between medication received by patients with the cognitive impairment.

Schizophrenia has been long recognized as a syndrome that subsumes several distinct illnesses, and the stratification of patients with schizophrenia keeps being an important goal. Clinical and pathophysiological markers are needed to specify treatment-relevant subgroups. For example, negative symptom subgroups that differ in their demographic, symptomatic, neuropsychological, and functional profiles have been proposed (49). Finding more homogenous subtypes may facilitate research and understanding of disease pathophysiology and more targeted interventions. Researchers are trying to derive clinical subgroups based on the severity of symptom burden (severe, predominantly positive, mild) (50) or trying to combine genetic and clinical data for subtyping predicting outcomes including treatment response, disease course and symptom severity using machine learning models (51). Although current findings do not support the notion of a major role for inflammation in cognitive impairment in schizophrenia, the possibility of an “inflammatory subtype” of schizophrenia has been proposed in which peripheral biomarkers of inflammation might strongly correlate within this subgroup of patients only (19). The long-term consequences of elevated levels of CRP require further investigation. Multi-center study in France found that peripheral low-grade inflammation (assessed by hs-CRP measurement) is associated with ultra-resistance to treatment in schizophrenia (52). Using CRP as a biomarker of peripheral inflammation in persons with schizophrenia may help to identify vulnerable patients and those that may benefit from adjunctive anti-inflammatory treatments (46). In the meantime, further research is needed to assess the effectiveness and safety of the neuroprotective anti-inflammatory strategies in order to prevent cognitive impairment in schizophrenia (42).

Findings of the current study should be analyzed in light of its limitations. First, the sample size is relatively small and reduces the power of a study. Second, it is a cross-sectional study and biomarkers levels were detected during a single examination of patients, and since this indicator is very labile, it can be assumed that in other patients it increased in certain periods of the disease. Because the subjects were in remission, the study could not address the issue of whether the biomarkers of interest are state-dependent or trait-dependent. The duration of untreated psychosis that could affect planning/problem-solving ability according to the meta-analysis (53) could not be accurately measured. Also, 86% of patients were treated with atypical antipsychotics that are associated with metabolic alterations, and metabolic syndrome has been shown to be associated with lower processing speed, attention and working memory (54). Although second generation atypical antipsychotics have been found to directly reduce expression and secretion of inflammatory cytokines in human immune independent from metabolic side effects and disease status (55), there is an association between atypical antipsychotics and changes in gut microbiota that can exacerbate inflammation through the immune system, increasing intestinal permeability, IL-1, IL-6 and CRP, which seem to affect cognitive functioning (56, 57).

## Conclusions

Schizophrenia has been long recognized as a syndrome that subsumes several distinct illnesses, and the stratification of patients with schizophrenia keeps being an important goal. Finding more homogenous subtypes may facilitate research and understanding of disease pathophysiology and more targeted interventions. Our findings further contribute to the accumulation of data on the possible role of the inflammation in the development of cognitive impairment in schizophrenia, confirming the CRP as the most promising biomarker among examined. CRP levels were predicted by the number of psychoses experienced (FES vs. MES), as well as cognitive functioning and PSP results.

## Data Availability Statement

The raw data supporting the conclusions of this article will be made available by the authors, without undue reservation.

## Ethics Statement

The studies involving human participants were reviewed and approved by Ethical Committee of Saint Petersburg State University. The patients/participants provided their written informed consent to participate in this study.

## Author Contributions

NP: project supervision, conceptualization, and funding acquisition. EC, MD, and NP: methodology and writing—original draft. EC, MD, and KT: investigation and data collection. EC and MD: statistical analyses and interpretation of results. MD, EC, KT, and NP: writing—review and editing. All authors contributed to the article and approved the submitted version.

## Funding

This work was supported by a grant of the Russian Scientific Foundation #14-50-0069 Translational Biomedicine at St. Petersburg State University.

## Conflict of Interest

The authors declare that the research was conducted in the absence of any commercial or financial relationships that could be construed as a potential conflict of interest.

## Publisher's Note

All claims expressed in this article are solely those of the authors and do not necessarily represent those of their affiliated organizations, or those of the publisher, the editors and the reviewers. Any product that may be evaluated in this article, or claim that may be made by its manufacturer, is not guaranteed or endorsed by the publisher.
