# Supplementary material for: A Cross-Sectional Study on Associations Between BDNF, CRP, IL-6 and Clinical Symptoms, Cognitive and Personal Performance in Patients With Paranoid Schizophrenia
Source: Front Psychiatry. 2022 Jul 6;13:943869. doi: 10.3389/fpsyt.2022.943869 (PMC9298757; doi:10.3389/fpsyt.2022.943869)
Supplement: Supplementary file 1 [file Table_1.docx]

Suppl. Table 1. Remission criteria according to Mosolov et al., 2014 (15).

| Dimensions of psychopathology | ICD-10 diagnosis (clinical forms) | F20.01 F20.03 | F20.00 | F20.02 |
| --- | --- | --- | --- | --- |
|  | PANSS items | Severity threshold score | | |
| Psychoticism (reality distortion) | Delusions (P1) | ≤3 | ≤5 | ≤3 |
|  | Unusual thought content (G9) | ≤3 | ≤5 | ≤3 |
|  | Hallucinations (P3) | ≤3 | ≤4 | ≤3 |
| Disorganization | Conceptual disorganization (P2) | ≤3 | ≤3 | ≤3 |
|  | Mannerisms/posturing (G5) | ≤3 | ≤3 | ≤3 |
| Negative symptoms | Blunted affect (N1) | ≤3 | ≤4 | ≤4 |
|  | Social withdrawal (N4) | ≤3 | ≤4 | ≤4 |
|  | Lack of spontaneity and flow of conversation (N6) | ≤3 | ≤4 | ≤3 |
| Autistic remission | Emotional withdrawal (N2) | – | ≤4 | ≤4 |
| Affective symptoms | Depression (G6) | ≤3 | ≤3 | ≤3 |
| Remission similar to that in acquired cyclothymia | Hyperactivity (P4) | ≤3 | – | ≤3 |
| Disturbance of volition | Disturbance of volition (G13) | ≤3 | – | ≤3 |
| Insight | Lack of judgment and insight (G12) | ≤3 | ≤4 | ≤3 |
| Supplemental aggression risk symptoms | Difficulties in delay of gratification (S2) | – | ≤4 | ≤4 |
|  | Affective lability (S3) | – | ≤4 | ≤4 |
| PSP scale |  | ≥71–80 | ≥51–60 | ≥51–70 |

ICD, International Classification of Diseases; PANSS, Positive and Negative Syndrome Scale; PSP, Personal and Social Performance Scale.
